# Stereotactic body radiation therapy for metastatic lung metastases

**DOI:** 10.1007/s11604-022-01323-9

**Published:** 2022-09-13

**Authors:** Tomoki Kimura, Toshiki Fujiwara, Tsubasa Kameoka, Yoshinori Adachi, Shinji Kariya

**Affiliations:** 1 Department of Radiation Oncology, Kochi Medical School, Kochi University, Oko-cho, Nangoku-shi, KohasuKochi, 783-8505 Japan; 2 Department of Radiation Oncology, Hiroshima Red Cross Hospital and Atomic-Bomb Survivors Hospital, 1-9-6 Sendamachi, Naka-ku, Hiroshima, 730-8619 Japan

**Keywords:** Stereotactic body radiation therapy (SBRT), Pulmonary oligometastatic disease (OMD)

## Abstract

Although systemic therapy is standard management for patients with metastatic disease, several recent reports have indicated that an addition of local therapies including stereotactic body radiation therapy (SBRT) for patients with oligometastatic disease (OMD) could improve survival. The lung is the most common site of distant metastasis from many solid tumors, and the strategy of SBRT, such as dose-fraction schedules, timing, etc., would be different depending on the type of primary tumor, location, and patterns of OMD. This review describes the role of SBRT with curative-intent for patients with pulmonary OMD for each of these variables. First, differences according to the type of primary tumor, for which many studies suggest that SBRT-mediated local control (LC) for patients with pulmonary OMD from colorectal cancer (CRC) is less successful than for those from non-CRC tumors. In addition, higher dose-fraction schedules seemed to correlate with higher LC; hence, different SBRT treatment strategies may be needed for patients with pulmonary OMD from CRC relative to other tumors. Second, differences according to location, where the safety of SBRT for peripheral pulmonary tumors has been relatively well established, but safety for central pulmonary tumors including pulmonary OMD is still considered controversial. To determine the optimal dose-fraction schedules, further data from prospective studies are still needed. Third, differences according to the patterns of OMD, the number of metastases and the timing of SBRT whereby 1–5 lesions in most patients and patients with synchronous or metachronous OMD are considered good candidates for SBRT. We conclude that there are still several problems in defining suitable indications for local therapy including SBRT, and that further prospective studies are required to resolve these issues.

## Introduction

According to the 8th Edition of the Union for International Cancer Control (UICC) TNM classification of lung cancer, single metastatic lesions in a single distant organ result in an M1b classification, because of the significant prognostic relevance of M1b staging versus multiple metastatic lesions in a single or multiple distant organs (M1c) [1]. Thus, a small number of metastases, such as reflected by M1b staging of lung cancer, are considered as causing oligometastatic disease (OMD), which is an intermediate state between localized and systemically metastasized disease [2]. Although systemic therapy is standard management for patients with metastatic disease, several recent reports suggest that an addition of local therapies including radiation therapy, such as stereotactic body radiation therapy (SBRT), for patients with OMD could improve survival [3–5]. However, OMD encompasses heterogeneous patients, and it remains unknown which patients would be eligible for local therapies. To proceed further, for prospective studies to determine eligibility for and the role of local therapies, the European Society for Radiotherapy and Oncology (ESTRO) and European Organisation for Research and Treatment of Cancer (EORTC) provided a classification of OMD in 2020 [6]. First, OMD is divided into two groups based on the history of polymetastatic disease, namely, genuine OMD (no history of polymetastatic disease) and induced OMD (previous history of polymetastatic disease). The latter refers to limited metastatic lesions for which local treatment is possible together with systemic chemotherapy when multiple metastases are present at the time of diagnosis. Genuine OMD is subclassified into de-novo OMD (first time diagnosis of OMD) and repeated OMD (previous history of OMD), which is defined as limited metastatic lesions newly re-progressing after local treatment for OMD. Finally, de-novo OMD is further subclassified into synchronous OMD, which is defined as limited metastatic lesions present at the same time, usually within about 6 months, and metachronous OMD, which is defined as limited metastatic lesions newly progressing after local treatment for the primary site, also usually after 6 months or more.

The lung is the most common site of distant metastasis from many solid tumors, such as primary lung cancer itself, colorectal cancer (CRC), head and neck cancer, renal cell cancer, breast cancer, soft tissue sarcoma, and others. About 30% of all patients with cancer will develop lung metastases at some point in the course of their disease [7]. Local therapies for patients with pulmonary OMD include resection, radiation therapy, and radiofrequency ablation. Although resection is recommended as the first choice of treatment for patients with pulmonary OMD, especially from CRC [8], radiation therapy also plays an important role as a local therapy. Curative radiation therapy is very similar to SBRT, the strategy for which, such as dose-fraction schedules, timing, etc., differs according to the type of primary tumor, location, and patterns of OMD. This review describes the role of curative-intent SBRT for patients with pulmonary OMD separately for each situation.

## Differences according to the primary tumor: non-lung primary malignancies or primary lung cancer

Treatment strategies for OMD are different for different primary tumors. Shultz et al. suggested that there are two scenarios, pulmonary metastases from non-lung primary malignancies and primary lung cancer [9].

### SBRT for pulmonary OMD from non-lung primary malignancies

**Fig. 1:**
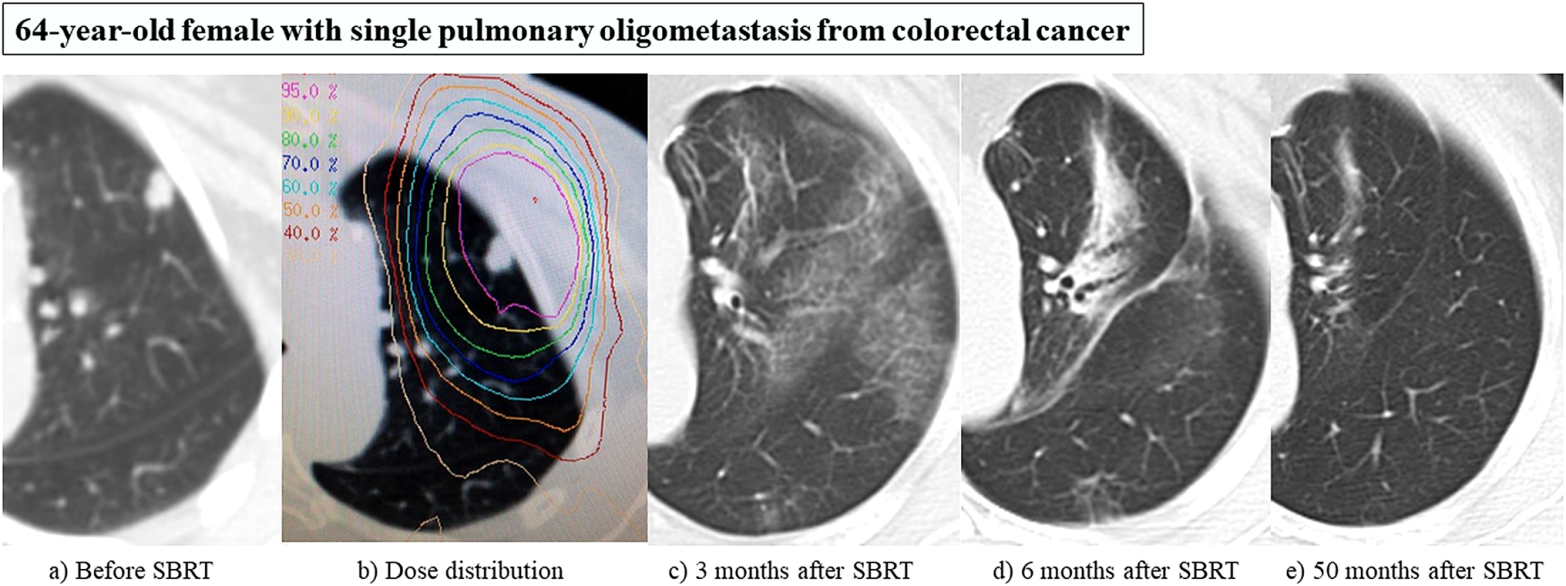
**a** 64-year-old female with single pulmonary oligometastasis from colorectal cancer. **a** Before SBRT. **b** Dose distribution: 48 Gy in 4 fractions, isocenter prescription (BED_10_ = 105.6 Gy). **c** 3 months after SBRT: Grade 1 radiation pneumonitis. **d** 6 months after SBRT: Post-irradiation change gradually shrinking. **e** 50 months after SBRT: Complete response

**Fig. 2:**
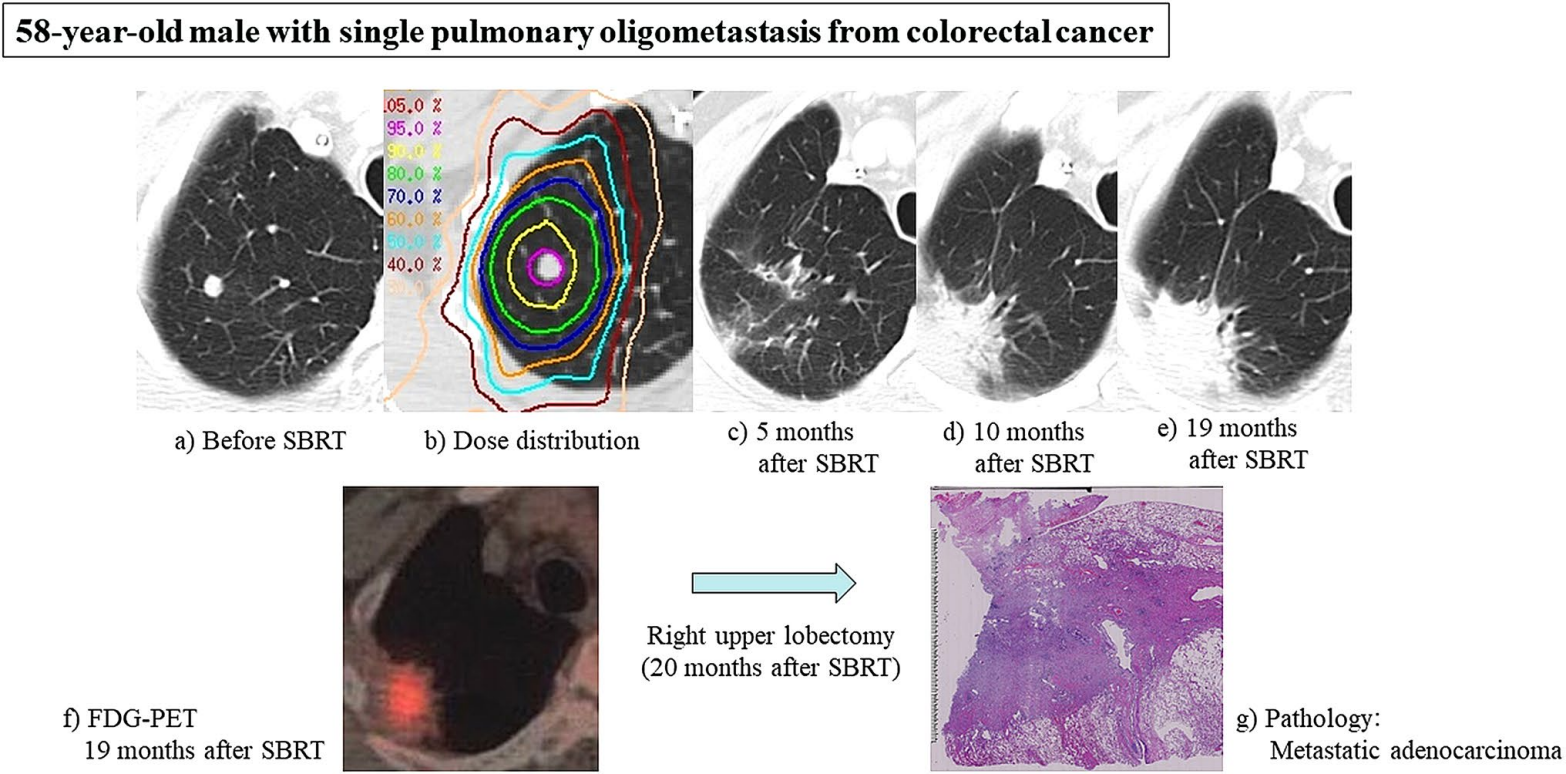
**a** 58-year-old male with single pulmonary oligometastasis from colorectal cancer. **a** Before SBRT. **b** Dose distribution: 56 Gy in 4 fractions, isocenter prescription (BED_10_ = 134.4 Gy). **c** 5 months after SBRT: Grade 1 radiation pneumonitis. **d** 10 months after SBRT: Post-irradiation change was mass-like appearance. **e** 19 months after SBRT: Mass-like appearance gradually increasing. **f** FDG-PET at 19 months after SBRT: Increased accumulation in mass-like appearance. **g** Pathology (right upper lobectomy in 20 months after SBRT9: Diagnosed as metastatic adenocarcinoma

Table 1 summarizes the outcome of SBRT for pulmonary OMD. CRC is the most common tumor of origin for pulmonary OMD from non-lung primary malignancies. Many authors reported differences in treatment results for pulmonary CRC-OMD compared with other tumors [10–24]. Several retrospective studies including only patients with pulmonary OMD from CRC showed that 2- or 3-year local control (LC) and overall survival (OS) rates were 60–70% and 50–64%, respectively, with low toxicities when using various different dose-fractionation schedules [14, 15, 17, 18]. Takeda et al. reported a retrospective comparison of CRC and non-CRC origins using the same dose of 50 Gy in five fractions, reporting a significant difference of 2 year LC (72% in CRC, 94% in non-CRC, *p* < 0.05) [12]. Helou et al. reported a prospective cohort study comparing the results of treating OMD of CRC-versus-non-CRC origins [19]. Although higher dose-fraction schedules were used for patients with CRC-OMD, LC was significantly lower than for OMD of non-CRC origin (2 year LC 76.4% in CRC, 91.7% in non-CRC, *p* < 0.001). Yamamoto et al. reported a large retrospective study of 1378 patients, indicating that LC of CRC origin OMD was also significantly lower for non-CRC (3 year LC 65.6% in CRC, 86.8% in non-CRC, *p* < 0.001) [23]. In addition, multivariate analysis of factors affecting LC showed that a CRC origin was significantly associated with worse LC. On the other hand, Guckenberger et al. found no significant difference in the likelihood of tumor control between pulmonary metastases from primary tumors of lung (148 patients), CRC (133 patients), or kidney (56 patients) origin [25]. Further prospective studies are needed, but in general, it can be said that LC of pulmonary OMD from CRC is worse than for non-CRC, according to the many studies shown in Table 1. Given that, what are the optimal dose-fraction schedules for pulmonary CRC-OMD? Takeda et al. reported that 2 year LC was 100% for 21 patients (12 liver, 9 lung) with CRC-OMD when using a higher dose-fraction schedule, such as a maximum dose of 83–100 Gy in 5 fractions (50–60 Gy in 5 fractions, 60% isodose) [26]. Helou et al. compared LC in 56 patients who received < 60 Gy in 4–5 fractions and 45 patients who received 60 Gy in 4 fractions and showed that delivering 60 Gy in 4 fractions was independently associated with a lower hazard of local failure (subdistribution hazard ratio 0.271, 95% confidence interval 0.078–0.940, *p* = 0.040) [19]. According to a systematic review of SBRT for oligometastatic CRC, in which the biological effective dose (BED) 10 (α/β = 10) ranged from 51.3 to 262.5 Gy, higher BED10 seems to correlate with higher LC [27]. Figures 1 and 2 show complete responses and local recurrence, respectively, after SBRT in patients with pulmonary CRC-OMD.

**Table 1:** SBRT for pulmonary oligometastatic disease (OMD)

Author/year	Study design	Patients	Lesions	(% CRC*)	Dose/fraction (Gy/fr)	Prescription	Local control	Overall survival	Toxicity grade≧3
Norihisa 2005, Japan [10]	Retrospective	34	43	26.5%	42–60 Gy/3 fr	Isocenter	90% (2y)	84.3% (2y)	3%
Rusthoven 2009, USA[11]	Phase I/II	38	63	23.7%	48- 60 Gy/ 3fr	80–90% isodose	96% (2y)	39% (2y)	7.9%
Takeda 2011, Japan [12]	Retrospective	34	44	CRC: 15 pts	50 Gy/ 5fr	80% isodose	CRC 72% (2y)	N.A	3%
				Non-CRC: 19 pts			Non-CRC 94% (2y)	N.A	
Widder 2013, Nertherlands [13]	Retrospective	42	N.A.**	73.8%	60 Gy/ 3fr	N.A	94% (2y)	62% (3y)	2.4%
Comito 2014, Italy [14]	Retrospective	41	60	100%	48–75 Gy/3-4fr	PTV D95％	70% (3y)	58% (3y)	0%
Jung 2015, Korea [15]	Retrospective	50	79	100%	48 Gy/4 fr (median)	85–90% isodose	70.6% (3y)	64% (3y)	0%
Rieber 2016, Germany [16]	Retrospective	700	N.A	21.9%	3–33 Gy × 1-13fr	88.7% isodose (median)	81.2% (2y)	54.4% (2y)	6.5%
Agolli 2016, Germany [17]	Retrospective	44	69	100%	23–45 Gy/1-3fr	95% isodose	60.2% (2y)	50.8% (3y)	0%
Jingu, 2017, Japan [18]	Retrospective	93	104	100%	40-65 Gy/3-15fr	Isocenter (83%)	65% (3y)	56% (3y)	2%
Helou, 2017, UK [19]	Prospective cohort	120	184	CRC: 101 pts	56-60 Gy/4fr	PTV D95%	76.4% (2y)	N.A	1.7%
				Non-CRC: 83 pts	48-52 Gy/4fr	PTV D95%	91.7% (2y)	N.A	
Osti, 2018, Italy [20]	Retrospective	129	166	31.7%	30 Gy/1fr	95% isodose	80.1% (3y)	34% (3y)	7.4%
Sharma, 2018, Nertherland [21]	Retrospective	206	327	57.3%	30-60 Gy/1-8fr	70–90% isodose	85% (2y)	36% (2y)	2%
Berkovic, 2020, Belgium [22]	Retrospective	104	132	33.7%	20-60 Gy/3 or 5fr	80% isodose	77.8% (3y)	72% (3y)	2%
Yamamoto 2020, Japan [23]	Retrospective	1378	1547	25.3%	48 Gy/4fr	Isocenter (71.3%)	81.3% (3y)	72% (3y)	2.5%
Siva 2021, Australia [24]	Randomized Phase II	45	69	46.7%	28 Gy/ 1fr	PTV D99%	64% (3y)	81% (3y)	5%
		45	69	46.7%	48 Gy/ 4fr	PTV D99%	80% (3y)	67% (3y)	3%

*CRC colorectal cancer, **NA not available, #Grade≧2, # PTV D95%/99%: the dose covering 95%/99% of the planning target volume (PTV)

### SBRT for oligometastatic lung cancer

The SBRT treatment strategy for pulmonary OMD from primary lung cancer is generally considered to be the same as for early-stage non-small cell lung cancer (NSCLC). For clinical stage IA (≦3 cm) NSCLC, the Japan Clinical Oncology Group (JCOG) 0403 Phase II trial showed that 3-year OS and LC was 76.5% and 85.4% in 64 operable patients, and 59.9% and 87.3% in 100 inoperable patients, with no severe toxicities when using 48 Gy in 4 fractions prescribed for the isocenter [28]. This dose-fraction schedule is considered equivalent to 42 Gy in 4 fractions prescribed as the dose covering 95% of the planning target volume (PTV) (D95%) using a superposition algorithm [29] and is the standard dose-fraction schedule for patients with c-stage IA NSCLC in Japan. For further improvement of treatment results and determination of the optimal dose-fraction schedule, a randomized Phase III trial (JCOG1408) is ongoing. This is comparing treatment outcomes of SBRT for patients with medically inoperable stage IA NSCLC and small lung lesions (tumor diameter within 3 cm) clinically diagnosed as NSCLC with 42 Gy in 4 fractions versus 55 Gy in 4 fractions at the D95%, 80% isodose of the PTV [30]. For c-T2N0M0 (tumor diameter 3–5 cm) NSCLC, a Phase I trial of SBRT, JCOG0702, recommended 55 Gy in 4 fractions for tumors with PTV < 100 cc [31], and 50 Gy in 4 fractions for tumors with PTV ≧100 cc [32]. For all c-T1-2 N0M0 NSCLC, National Comprehensive Cancer Network (NCCN) guidelines recommend several dose-fraction schedules, such as 25–34 Gy in one fraction, 45–60 Gy in 3 fractions, 48–50 Gy in 4 fractions and 50–55 Gy in 5 fractions, for peripheral tumors [33]. In Western countries, 54 Gy in 3 fractions is commonly used in routine practice and for clinical trials (RTOG0236/0618) [34, 35]. Another dose-fraction schedule, 34 Gy in one fraction, is also considered a good option (RTOG0915) [36]. Onishi et al. analyzed various dose-fraction schedules for 257 patients with c-stage I NSCLC and reported that the LC and survival rates were better with a BED_10_≧100 Gy than with < 100 Gy [37]. At least, a BED_10_≧100 Gy would be needed to achieve satisfactory LC and better survival [33].

### Impact of LC on survival

Theoretically, prevention of the proliferation of tumor cells and their invasion through adjacent tissues and basement membranes by LC could reduce the incidence of lymph node metastasis and distant metastasis in many primary cancers [37]. SBRT is one of the local therapies that help to achieve LC, and several reports on its use for primary lung cancer and pulmonary OMD indicate that better local control improves OS [23, 38, 39]. In a multivariate analysis, Yamamoto et al. showed that LC was one of the significant factors for OS in 1378 patients with pulmonary OMD who received SBRT [23]. In addition, several retrospective studies showed that higher SBRT doses could improve not only LC but also OS [38, 39]. Further prospective studies are warranted.

### Differences according to tumor location: peripheral-versus-central

The safety of SBRT for peripheral pulmonary tumors has been relatively clearly established, but for central pulmonary tumors, which are typically located close to organs such as the proximal bronchial tree, esophagus, or great vessels, it is still considered controversial. Several prospective studies of SBRT for centrally located stage I NSCLC have been reported. Thus, Kimura et al. reported the phase I study (JROSG10-1) that recommended a dose of 60 Gy in 8 fractions at the isocenter, which is considered equivalent to 50 Gy in 8 fractions prescribed at PTV D95%, without grade ≧3 adverse effects for patients with T1 (≦ 3 cm) N0M0 centrally located NSCLC [40]. Bezjak et al. reported a phase I/II study (RTOG0813) that recommended a dose of 60 Gy in 5 fractions prescribed at D95% with 7.2% grade ≧3 adverse effects for patients with T1 or 2 (≦ 5 cm) N0M0 centrally located NSCLC [41]. Patients in the high-dose group in that study (57.5 Gy or 60 Gy in 5 fractions) had a similar OS as patients with peripheral tumors with high rates of tumor control. However, considering that instances of fatal toxicity have been reported [42], the use of SBRT for central pulmonary tumors should be carefully considered. This applies even more to ultracentral pulmonary tumors, which are defined as lesions whose gross tumor volume (GTV) or PTV abuts the proximal bronchial tree and/or other mediastinal structures [43]. Hypofractionated schedules may be considered in these cases. Karasawa et al. reported that accelerated hypofractionated radiotherapy with 75 Gy in 25 fractions at the isocenter, which is considered equivalent to 62.5 Gy in 25 fractions prescribed at PTV D95%, is promising in that it can achieve LC and survival results similar to SBRT, and it can control both central and peripheral stage I NSCLC without any serious organ toxicities [44].

On the other hand, there is also no consensus on the application of SBRT for centrally located pulmonary OMD. Table 2 shows the details of several studies [21, 45–51]. The number of fractions tended to increase (5–10 fractions) compared to the SBRT for peripheral OMD, as shown in Table 1. In addition, the incidence of grade 5 toxicities also tended to increase. Lindberg et al. reported the HILUS Trial, a prospective Nordic multicenter phase II study of ultracentral lung tumors treated with SBRT of 56 Gy in 8 fractions, prescribed to the PTV-encompassing isodose [51]. Of a total of 65 patients, grade 3 to 5 toxicity was observed in 22 (34%), including 10 cases of treatment-related death (15%). Therefore, the authors concluded that this dose-fraction schedule should not be used for tumors located within 1 cm of the main bronchi and trachea. According to the American Society for Radiation Oncology (ASTRO) guidelines, when using SBRT for centrally located tumors, physicians should endeavor to meet the dose constraints that have been utilized in prospective or other studies, given the severe toxicities that have been reported [52]. To determine the optimal dose-fraction schedules, further data from prospective studies are needed.

**Table 2:** SBRT for centrally located pulmonary oligometastatic disease (OMD)

Author/year	Study design	Patients	Lesions	Lesions of OMD (%)	Dose/fraction (Gy/fr)	Prescription	Local control	Overall survival	Toxicity grade≧3 (Grade 5)
Milano 2009, USA [45]	Retrospective	53	63	34 (54)	30–60 Gy/ 4–18 fr	80% isodose	73% (2y)	44% (2y)	N.A. (19%)
Rowe 2012, USA [46] (USA, IN)	Retrospective	47	51	21 (41%)	50 Gy/ 4fr	70–90% isodose	94% (2y)	N.A*	13% (2%)
Davis, 2015, USA [47]	Retrospective	64	66	66 (100%)	37.5 Gy/ 3fr (median)	N.A	69.8% (2y)	49.6% (2y)	0%
Lischalk, 2016, USA [48]	Retrospective	20	20	20 (100%)	35 or 40 Gy/ 5fr	PVT D95%**	57.4% (2y)	40% (2y)	10% (0%)
Figlia, 2018, Irtaly [49]	Retrospective	39	39	13 (33%)	40–70 Gy/ 8–10 fr	PVT D95%	92.9% (2y)	83.9% (2y)	0%
Chang, 2018, Australia [50]	Retrospective	107	107	107 (100%)	30–50 Gy1-3 fr	83% isodose	96.6%/ 95.7% (2y)#	55.1% (2y)	5.6% (2.8%)
Sharma, 2018, Netherland [21]	Retrospective	N.A	83	83 (100%)	45-60 Gy/5fr	70–90% isodose	82% (3y)	N.A	2% (0%)
Lindberg, 2021, Sweden [51]	Phase II	65	68	14 (22%)	56 Gy/8fr	67% isodose	83% (3y)	50% (3y)	34% (15%)

*N.A not available, **D95%: the dose covering 95% of the planning target volume (PTV), # 96.6% in central tumors and 95.7% in ultracentral tumors trails of

## Differences according to the patterns of OMD: the number of metastases and the timing of SBRT

Eligibility criteria for applying SBRT for pulmonary OMD are considered to include operability, tumor location, number of metastases and the timing of treatment. The tumor location, such as peripheral or central, has already been discussed above. Operability is judged by pulmonary function, comorbidities, previous history of thoracic resection, and other factors in the same manner as early-stage lung cancer. Regarding the number of metastases and the timing of treatment, several randomized phase II studies, which compared local therapies including SBRT and maintenance therapies or observation for patients with OMD, provide us with useful information [3–5] (Table 3). These trials showed that local therapies including SBRT improved progression-free survival or OS compared with maintenance therapies. The eligibility criteria determining the number of metastases were three metastatic sites plus primary NSCLC in Gometz’s study [3], five metastatic sites plus primary NSCLC in Iyengar’s study [4] and five metastatic sites within three organs plus various primary tumors in SABR-COMET [5], respectively. According to the European Society for Radiotherapy and Oncology (ESTO)-American Society for Radiation Oncology (ASTRO) consensus document, OMD was defined as a limited number of metastases, 3 to 5, or less [53]. In fact, the results of these studies showed that the number of metastases was 1 to 3 lesions in most of the patients.

**Table 3:** SBRT for pulmonary oligometastatic disease (OMD)

Author/year	Primary	Eligibility	Patients	Number of lung lesions	Dose/fractions for lung lesions (Gy)	OS	PFS	Treatment-related death
Gometz 2016, USA	Stage IV NSCLC	Stable with 4≧cycles first-line chemotherapy or 3≧ months of EGFR^✝^/ALK^¶^ inhibitors. Primary plus up to 3 metastatic sites	49 (Local therapy: 25 vs maintenance: 24)	Local therapy: 27 vs maintenance: 27	5 lesions: 50 Gy/4fr	N.A.**	MST: 11.9 M vs 3.9 M (*p* = 0.0054)	None
					3 lesions: 52.5 Gy/15fr, 66 Gy/30fr			
					2 lesions: 60 Gy/30fr, 70 Gy/10fr, 45 Gy/15fr			
					1 lesion: 66 Gy/33fr, 60 Gy/15fr, 67.5 Gy/27fr, 60 Gy/8fr, 48 Gy/4fr, 55 Gy/15fr			
Iyengar, 2017, USA	Stage IV NSCLC	Stable with 4 to 6 cycles first-line chemotherapy (EGFR/ALK inhibitors were excluded). Primary plus up to 5 metastatic sites	29 (SBRT:14 vs maintenance: 15)	SBRT:16 vs maintenance: 18	10 lesions: 18–21 Gy/1fr	N.A	MST: 9.7 M vs 3.5 M (*p* = 0.01)	None
					5 lesions: 45 Gy/15fr			
					1 lesion: 33 Gy/3fr			
Palma 2020, Canada	Lung:18 ptsBreast 18 pts. CRC: 18 pts. Prostate: 16 pts. Others: 29 pts	Stable with standard systemic therapy for 3 months or more. Controlled primary plus up to 5 metastatic sites (within 3 organs)	99 (SBRT:66 vs maintenance: 33)	SBRT: 55 vs maintenance: 34	54 Gy/3fr, 55 Gy/5fr, 60 Gy/8fr	42.3% vs 17.7% (5y) (*p* = 0.006)	17.3% vs 3.2% (5y) (p = 0.001)	3 pts (4.5%) in SBRT

*including primary lung cancer, **N.A not available, ^✝^EGFR epidermal growth factor receptor, ^¶^ *ALK* anaplastic lymphoma kinase

**Fig. 3 A:**
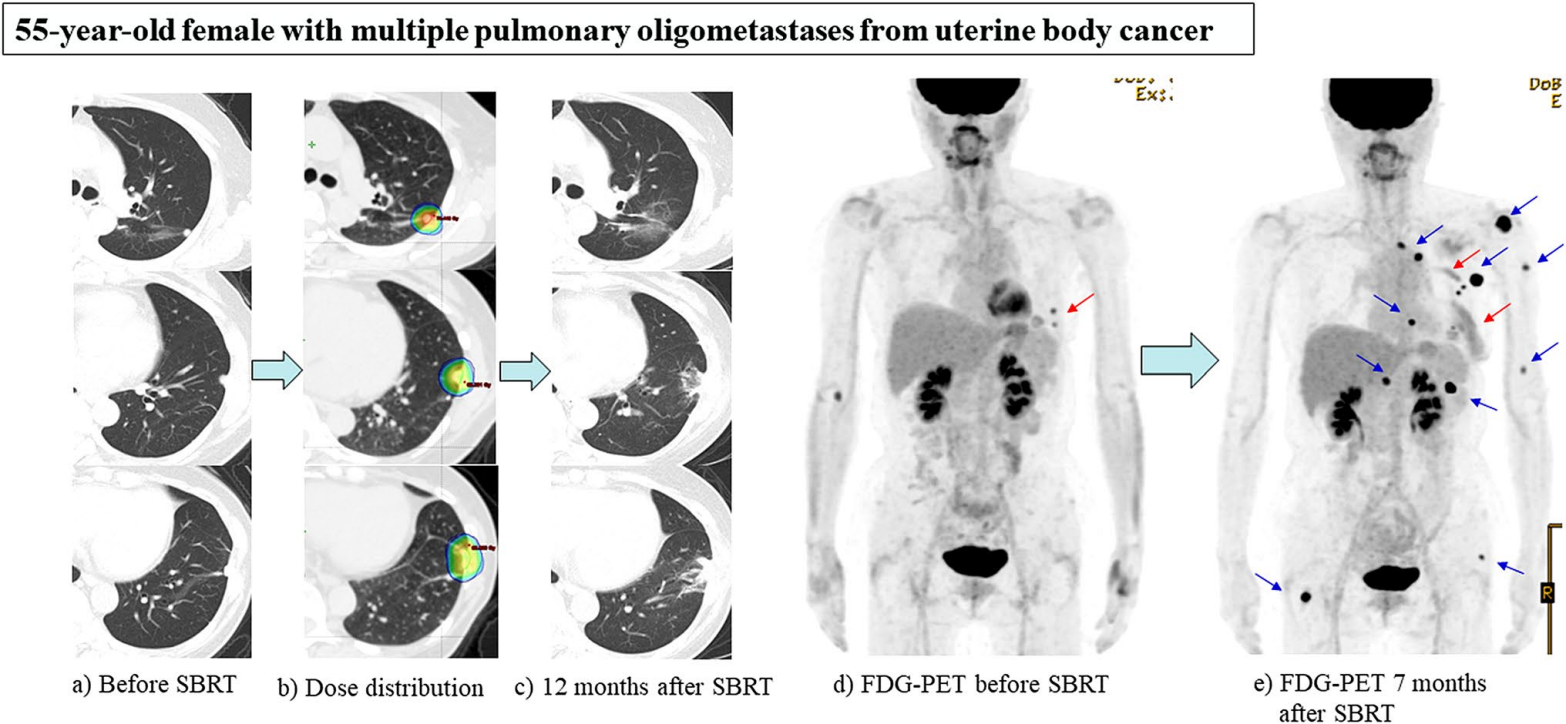
55-year-old female with multiple pulmonary and pleural oligometastases from uterine body cancer. This patient had repeatedly undergone resection for pulmonary metachronous oligometastatic disease (OMD), but three pulmonary and pleural metastases rapidly recurred after resection resulting in a diagnosis of repeated OMD. **a** Before SBRT. **b** Dose distribution: 56 Gy in 4 fractions, D95% prescription, 80% isodose (BED_10_ = 134.4 Gy, BED_10max_ = 192.5 Gy). **c** 12 months after SBRT: Post-irradiation change (Grade 1 radiation pneumonitis). Diagnosed as local control. **d** FDG-PET before SBRT: Accumulation at two pulmonary and pleural oligometastases (red arrow). **e** FDG-PET at 7 months after SBRT: Although the three irradiated lesions were controlled locally (red arrow), multiple metastases at axillary lymph node, skin and lungs were observed at the same time 7 months after the completion of SBRT (blue arrow)

What of the timing of treatment? In general, the timing of local therapies has not been established obviously. The trials shown in Table 3 were considered as investigating synchronous OMD. For patients with synchronous OMD, local therapies including SBRT are usually intervened after the completion of the first-line chemotherapy. De-novo metachronous OMD is also a good candidate for SBRT because most patients are administered curative treatment for the primary tumor after 6 months or more. In particular, the goal of SBRT for metachronous rather than synchronous OMD is considered to be to achieve control of metastatic sites [53]. For patients with repeated OMD, SBRT is also one of the possible local therapies. The timing of intervention of SBRT for the repeated OMD is usually considered at the time when new OMD is found as well as that of metachronous OMD. However, the importance of the maintenance treatment after the completion of SBRT for repeated OMD would increase comparing to that of metachronous OMD. Figure 3 shows a case of SBRT for repeated OMD. This patient had repeatedly undergone resection for pulmonary metachronous OMD, but three pulmonary and pleural metastases rapidly recurred after resection and the patient was diagnosed as having repeated OMD. Although SBRT was performed for these lesions and controlled the disease locally, multiple metastases at axillary lymph nodes, skin and lungs were observed at the same time 7 months after completion of SBRT. It is important that local therapies including SBRT should be applied before repeated OMD develops into polymetastatic disease, as is the case for induced OMD. The timing of intervention using local therapies for patients with repeated or induced OMD has to be judged individually after careful consideration in each case. In addition, the trials shown in Table 3 focused on the addition of local therapies including SBRT, but the combination of SBRT and immune checkpoint inhibitors (ICI) was not referred. Sharabi et al. described that SBRT can produce immune-mediated systemic responses and induce an "abscopal effect", therefore, SBRT, combined with ICI, increases tumor cell susceptibility to immune-mediated cell death. [54]. In a clinical setting, Tang et al. reported a phase I trial testing SBRT with cytotoxic T lymphocyte antigen 4 (CTLA-4) and ipilimumab for patients with metastatic solid tumors of the liver or lung refractory to standard therapies. They concluded that combining SBRT and ipilimumab was safe with a 10% partial response in non-irradiated lesions, and irradiation to the liver produced greater T-cell activation than did irradiation to the lung [55]. From these evidences, the combination of SBRT and ICI could improve survival more than SBRT alone for patients with OMD.”

For multiple pulmonary lesions, the optimal dose-fraction schedules of curative-intent SBRT remain controversial. Table 3 also shows the various dose-fraction schedules for 1 to 5 metastatic lesions plus primary site, which would be considered as radical dose setting. Kobiela et al. described that a higher number of lesions might correlate with lower LC and, therefore, OS in their systematic review of SBRT for pulmonary OMD from CRC [56]. Chmura et al. reported results of the NRG-BR001 phase I trial, which was to establish safety of SBRT dose-fraction schedules in patients with 3–4 metastases or 2 metastases in close proximity to each other [57]. In that trial, 45 Gy in 3 fractions and 50 Gy in 5 fractions were used for peripheral and centrally located pulmonary OMD, respectively. There was no dose-limiting toxicity and the authors concluded that these dose-fraction schedules could be recommended for patients with multiple metastases with acceptable short-term toxicities using curative-intent SBRT developed for a single metastasis or primary tumors. On the other hand, Palma et al. suggested that the goal of using SBRT for all lesions should not be cure, but merely a temporary tumor growth arrest with minimization of toxicities, an approach they called “Ablative Radiation Therapy to Restrain Everything Safely Treatable (ARREST)” [58]. In fact, the dose-fraction schedules of 54 to 60 Gy in 3 to 8 fractions were recommended for pulmonary OMD in the SABR-COMET trial [5], but these have been reduced to doses of 20 to 35 Gy in 1 to 5 fractions in the ongoing SABR-COMET-10 trial, which treats 4–10 oligometastatic tumors [59]. However, the evaluation of this ARREST concept has just started by Phase I study [60], and there is no evidence at this moment. We should aim to cure the limited number of OMD using curative-intent SBRT to improve the survival for patients with OMD, as described this manuscript.

Finally, what is the expected benefit of SBRT for patients with pulmonary OMD? Lehrer et al. reported the results of a meta-analysis of 21 studies comprising 943 patients with 1290 oligometastases (≦5 lesions of extracranial disease), for which SBRT was administered in ≦8 fractions with ≧5 Gy per fraction [61]. The lesions treated by SBRT included those in the lung in 29.2% of patients. One-year LC and OS rates were 94.7% (95% CI 88.6–98.6%) and 85.4% (95% CI 77.1–92.0%), respectively. Acute and late grade 3 to 5 toxicities occurred in 1.2% (95% CI 0–3.8%) and 1.7% (95% CI 0.2–4.6) of cases, respectively. Ongoing and planned prospective studies are needed to confirm these results.

## Conclusions

This review discussed different strategies for employing SBRT for patients with pulmonary OMD according to the type of primary tumor, location, and patterns of OMD. Considering these differences, it is concluded that curative-intent SBRT for limited pulmonary OMD does offer the possibility of improving not only LC but also OS, according to the results of the studies reviewed here. Recently, indications for different treatment strategies for OMD including pulmonary lesions are expanding, such as for treating increasing numbers of lesions, due to the development of radiation therapy and ICI therapies. Therefore, the goal of SBRT may be changing from curative intent to the aim of temporarily causing tumor growth arrest with minimization of toxicities. There are still several problems for determining the most appropriate indications for local therapy interventions, and further prospective studies are expected to resolve these issues.
