# Correction to: Stereotactic body radiation therapy for metastatic lung metastases

**DOI:** 10.1007/s11604-022-01342-6

**Published:** 2022-09-26

**Authors:** Tomoki Kimura, Toshiki Fujiwara, Tsubasa Kameoka, Yoshinori Adachi, Shinji Kariya

**Affiliations:** 1 Department of Radiation Oncology, Kochi Medical School, Kochi University, Oko-cho, Nangoku-shi, KohasuKochi, 783-8505 Japan; 2 Department of Radiation Oncology, Hiroshima Red Cross Hospital and Atomic-Bomb Survivors Hospital, 1-9-6 Sendamachi, Naka-ku, Hiroshima, 730-8619 Japan

## Correction to: Japanese Journal of Radiology 10.1007/s11604-022-01323-9

In the original publication, Tables 1 and 2 were published incorrectly.

The correct version of Tables 1 and 2 is given in this Correction.

**Table 1:** SBRT for pulmonary oligometastatic disease (OMD)

Author/year	Study design	Patients	Lesions	Primary cancer (% CRC*)	Dose/fraction (Gy/fr)	Prescription	Local control	Overall survival	Toxicity grade ≧ 3
Norihisa, 2005, Japan [10]	Retrospective	34	43	26.5%	42–60 Gy/3 fr	Isocenter	90% (2y)	84.3% (2y)	3%
Rusthoven, 2009, USA [11] (USA, IN)	Phase I/II	38	63	23.7%	48- 60 Gy/3 fr	80–90% isodose	96% (2y)	39% (2y)	7.9%
Takeda, 2011, Japan [12]	Retrospective	34	44	CRC: 15 pts	50 Gy/5 fr	80% isodose	CRC 72% (2y)	N.A	3%
				Non-CRC: 19 pts			Non-CRC 94% (2y)	N.A	
Widder, 2013, Nertherlands [13]	Retrospective	42	N.A.**	73.8%	60 Gy/3 fr	N.A	94% (2y)	62% (3y)	2.4%
Comito, 2014, Italy [14]	Retrospective	41	60	100%	48–75 Gy/3-4fr	PTV D95%^#^	70% (3y)	58% (3y)	0%
Jung, 2015, Korea [15]	Retrospective	50	79	100%	48 Gy/4 fr (median)	85–90% isodose	70.6% (3y)	64% (3y)	0%
Rieber, 2016, Germany [16]	Retrospective	700	N.A	21.9%	3–33 Gy × 1–13 fr	88.7% isodose (median)	81.2% (2y)	54.4% (2y)	6.5%^#^
Agolli, 2016, Germany [17]	Retrospective	44	69	100%	23–45 Gy/1–3 fr	95% isodose	60.2% (2y)	50.8% (3y)	0%
Jingu, 2017, Japan [18]	Retrospective	93	104	100%	40–65 Gy/3–15 fr	Isocenter (83%)	65% (3y)	56% (3y)	2%
Helou, 2017, UK [19]	Prospective cohort	120	184	CRC: 101 pts	56–60 Gy/4 fr	PTV D95%	76.4% (2y)	N.A	1.7%
				Non-CRC: 83 pts	48–52 Gy/ 4 or 5 fr	PTV D95%	91.7% (2y)	N.A	
Osti, 2018, Italy [20]	Retrospective	129	166	31.7%	30 Gy/1 fr	95% isodose	80.1% (3y)	34% (3y)	7.4%
Sharma, 2018, Netherland [21]	Retrospective	206	327	57.3%	30–60 Gy/1–8 fr BED	70–90% isodose	85% (2y)	36% (2y)	2%
Berkovic, 2020, Belgium [22]	Retrospective	104	132	33.7%	20–60 Gy/3 or 5 fr	80% isodose	77.8% (3y)	72% (3y)	2%
Yamamoto, 2020, Japan [23]	Retrospective	1378	1547	25.3%	48 Gy/4 fr (median)	Isocenter (71.3%)	81.3% (3y)	60.3% (3y)	2.5%
Siva, 2021, Australia [24]	Randomized Phase II	45	69	46.7%	28 Gy/1 fr	PTV D99%#	64% (3y)	81% (3y)	5%
		45	69	46.7%	48 Gy/4 fr	PTV D99%	80% (3y)	67% (3y)	3%

*CRC: Colorectal cancer, **N.A.: not available, #Grade ≧ 2, # PTV D95%/99%: the dose covering 95%/99% of the planning target volume (PTV)

**Table 2:** SBRT for centrally located pulmonary oligometastatic disease (OMD)

Author/year	Study design	Patients	Lesions	Lesions of OMD (%)	Dose/fraction (Gy/fr)	Prescription	Local control	Overall survival	Toxicity grade ≧ 3 (Grade 5)
Milano, 2009, USA [45]	Retrospective	53	63	34 (54%)	30–60 Gy/ 4–18 fr	80% isodose	73% (2y)	44% (2y)	N.A. (19%)
Rowe, 2012, USA [46] (USA, IN)	Retrospective	47	51	21 (41%)	50 Gy/4 fr	70–90% isodose	94% (2y)	N.A.*	13% (2%)
Davis, 2015, USA [47]	Retrospective	64	66	66 (100%)	37.5 Gy/3 fr (median)	N.A	69.8% (2y)	49.6% (2y)	0%
Lischalk, 2016, USA [48]	Retrospective	20	20	20 (100%)	35 or 40 Gy/5 fr	PTV D95%**	57.4% (2y)	40% (2y)	10% (0%)
Figlia, 2018, Italy [49]	Retrospective	39	39	13 (33%)	40–70 Gy/8–10 fr	PTV D95%	92.9% (2y)	83.9% (2y)	0%
Chang, 2018, Australia [50]	Retrospective	107	107	107 (100%)	30–50 Gy/1–3 fr	83% isodose	96.6%/95.7% (2y)^#^	55.1% (2y)	5.6% (2.8%)
Sharma, 2018, Netherland [21]	Retrospective	N.A	83	83 (100%)	45–60 Gy/5 fr	70–90% isodose	82% (3y)	N.A	2% (0%)
Lindberg, 2021, Sweden [51]	Phase II	65	68	14 (22%)	56 Gy/8 fr	67% isodose	83% (3y)	50% (3y)	34% (15%)

*N.A.: not available, ** PTV D95%: the dose covering 95% of the planning target volume (PTV), # 96.6% in central tumors and 95.7% in ultracentral tumors

The original publication has been corrected.

